# Determination of Deamidation in Adjuvanted Vaccine Antigens through Isoaspartic Acid Quantification

**DOI:** 10.3390/vaccines12070733

**Published:** 2024-07-02

**Authors:** Manvi Hasija, Jian Ma, Bing Li, Nausheen Rahman, Kirsten A. Strahlendorf, Salvador Fernando Ausar

**Affiliations:** Global BioProcess Development, Sanofi, 1755 Steeles Ave West, Toronto, ON M3R 3T4, Canada; maj15@mcmaster.ca (J.M.); bing.li1@sanofi.com (B.L.); nausheen.rahman@sanofi.com (N.R.); kirsten.strahlendorf@sanofi.com (K.A.S.)

**Keywords:** vaccine, stability, deamidation, formulation, isoaspartic acid, degradation

## Abstract

Deamidation is a post-translational chemical modification that occurs within proteins and can be influenced by many factors, including temperature and pH. In vaccines, deamidation is considered undesirable as it may lead to changes in structure, function, stability, and immunogenicity. Detecting deamidation in vaccines, especially adjuvanted vaccines, can be challenging due to the lack of simple quantitative techniques. In this study, the quantification of isoaspartic acid (isoAsp) was used to assess deamidation in model antigens in the presence and absence of common vaccine adjuvants. This study shows that the detection of isoAsp was possible in the presence of various types of adjuvants with little to no interference. High levels of isoAsp were detected in thermally and pH-stressed adjuvanted vaccines, suggesting significant deamidation and highlighting the stability-indicating capabilities of the assay. The quantification of isoAsp in stability programs of a vaccine drug product could possibly find applications in product shelf-life determination, using thermal kinetic modeling to predict deamidation over time. The ability to detect deamidation early in vaccine development enhances process improvements and ultimately improves the vaccine’s stability. To summarize, this paper describes a rapid and simple method to determine deamidation in adjuvanted vaccines. This method could be applicable to formulation development, stability assessment, or shelf-life determination.

## 1. Introduction

Vaccines are biological products with compositions that can range from a single protein or nucleic acid to more complex entities such as polysaccharide–protein conjugates, virus-like particles (VLPs), outer membrane vesicles (OMVs), and attenuated microorganisms [1]. An added complexity of vaccine formulations is the presence of adjuvants, which are often added to enhance the immunogenicity of purified antigens [2]. Several types of adjuvants are either on the market or under development, including aluminum salts, oil emulsions, toll-like receptor agonists, synthetic DNA, immune-stimulating complexes (ISCOM), and antibacterial peptides, to mention a few. Many of these compounds interact with antigens and could exert stabilizing or, sometimes, destabilizing effects, depending on the solution conditions [3]. During bioprocessing, storage, and distribution, regardless of the presence or absence of an adjuvant, vaccine products can be exposed to various stress-inducing conditions, leading to instability and a loss in potency. Understanding the major pathways of degradation is important for designing formulation strategies that could prevent the instability of each vaccine component [4]. Due to their inherited structural complexity, vaccines are susceptible to physical and chemical degradation [1,5]. Physical-based mechanisms of degradation may include a variety of alterations in the biomolecule’s structure, including, but not limited to, unfolding, aggregation, and precipitation. Chemically induced degradation, on the other hand, involves any process that leads to the breaking or formation of new covalent bonds and the generation of new chemical species. While these two major mechanisms of degradation can be categorized distinctly, it is important to recognize that they are intricately linked, often giving rise to synergistic effects in the context of protein stability [4,5].

Together with oxidation, deamidation and isomerization are the most common routes of chemical degradation in proteins [6]. In particular, the asparagine (Asn) deamidation and aspartate (Asp) isomerization are the causes of major instability in protein formulations at neutral and alkaline pH conditions [7,8]. Protein deamidation is a chemical reaction in which an amide functional group is removed from an organic compound. In protein antigens, this reaction is crucial as the conversion of amide-containing side chains of amino acids can lead to conformational changes in the protein antigens [4,6,9]. Similarly, degradation through isomerization occurs when Asp converts into isoaspartic acid (isoAsp) [7]. The degradation of antigens through protein deamidation has been shown to reduce the efficacy of the vaccine by reducing the immunogenicity against the deamidated protein antigen. The best example of this is the spontaneous deamidation reported in multiple Asn residues of recombinant protective antigen (rPA) used to prepare anthrax vaccines, leading to decreased immunogenicity [9,10]. Thus, understanding protein deamidation/isomerization in vaccines could potentially lead to improved long-term efficacy and storage times for vaccines. 

A wide variety of tools have been used to identify and assess chemical degradation of vaccine antigens. Sodium dodecyl sulfate–polyacrylamide gel electrophoresis (SDS-PAGE) can be used to determine large changes in protein size; however, it cannot accurately determine small mass changes such as those involved in protein deamidation [2,4,5]. Currently, protein mapping followed by mass spectrometry (MS) is one of the approaches to protein deamidation determination that is widely used in the vaccine industry [6]. This technique has been extensively used to detect and characterize changes in specific chemical groups. Additionally, this technique can detect the occurrence of protein deamidation but does not allow quantifying the extent of that degradation. In addition, to make things even more complex, adjuvant components often interfere with liquid chromatography–mass spectrometry (LC-MS) and require sophisticated sample preparation protocols to assess protein deamidation [6,11].

In this study, we have evaluated the ability of ISOQUANT^®®^ Isoaspartate Detection Kit to quantify the degree of deamidation of a variety of antigens. This technique can measure deamidation by using protein isoaspartyl methyltransferase (PIMT), which can detect the presence of isoAsp and quantify the amount of isoAsp present within a sample [12,13]. In the same way, this system is also able to identify the amount of IsoAsp caused by protein isomerization; however, it is not able to distinguish if the isoAsp originated from deamidation or isomerization [12,13]. In this study, the isoAsp detection method will be used to detect deamidation in thermally stressed vaccine protein and virus-based drug products. Furthermore, the evaluation of the isoAsp detection method’s ability to determine the deamidation rate in adjuvanted vaccine products was studied. Finally, the isoAsp detection method was used to quantify vaccine deamidation in adjuvanted vaccine drug products under different pH and to determine the shelf-life of samples under different conditions.

## 2. Materials and Methods

### 2.1. Materials and Formulation

The model antigens, pneumococcal choline-binding protein A (PcpA) (MW = 49 kDa) and a recombinant fusion protein (RFP) (MW = 41.3 kDa), were expressed in Escherichia coli and purified by column chromatography, yielding a purity higher than 90%, as evaluated by SDS-PAGE. Protein stock solutions were supplied at approximately 1 mg/mL in 10 mM Tris, pH 7.4, containing 150 mM sodium chloride (TBS). 

Inactivated poliovirus (IPV) type 2 stock was supplied in phosphate buffer pH 7.4. Aluminum hydroxide (AlOOH) adjuvant (Alhydrogel^®®^) was obtained from Croda (Denmark). Squalene emulsion (AF03) and a two-component adjuvant consisting of a toll-like receptor 9 agonist and a cationic peptide acting as a vehicle (referred to as TLR-9 throughout the manuscript) adjuvant were produced as described elsewhere [14,15]. All other chemicals were analytical-grade reagents.

Phosphate-treated aluminum hydroxide (PTAH) was prepared by adding 0.4 M phosphate buffer pH 7.4 to a stock solution of AlOOH and mixing for 30 min at room temperature. The final concentration of aluminum was 560 μg/mL, and the final concentration of phosphate buffer was 20 mM to reach a phosphate/aluminum (P/Al) molar ratio of 1.0 (PTAH-1.0).

Thermally stressed RFP samples were obtained after 26 weeks of incubation at 37 °C. Thermally stressed IPV and PcpA were obtained after 2 weeks of incubation at 45 °C. 

### 2.2. Quantitation of IsoAsp Residue by Reversed-Phase High-Performance Liquid Chromatography

**Figure 1:**
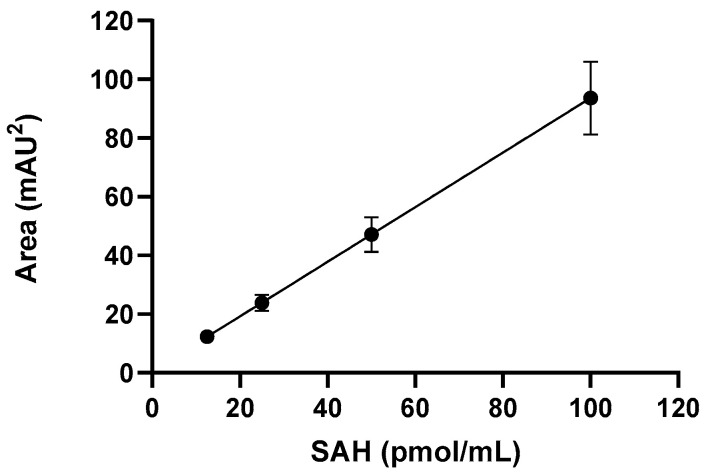
Calibration curve produced with S-Adenosyl homocysteine (SAH). Error bars represent the standard deviation from the mean (*n* = 3).

The ISOQUANT Isoaspartate Detection Kit (Promega, Madison, WI, USA) was used to quantify the formation of isoAsp resulting from deamidation. This method employs PIMT to specifically detect the presence of isoAsp residues in a target protein resulting from the gradual non-enzymatic deamidation of asparagine or rearrangement of aspartic acid residues during storage or handling. PIMT catalyzes the transfer of a methyl group from S-adenosyl-L-methionine (SAM) to isoAsp at the a-carboxyl position, generating S-Adenosyl homocysteine (SAH) in the process. SAH, a relatively small molecule, is quantitated by reversed-phase high-performance liquid chromatography (RP-HPLC) in an Agilent 1200 HPLC system equipped with a diode array UV detector. Samples were incubated with PIMT and SAM in sample buffer for 30 min at 30 °C, and the methylation reaction was terminated with stop solution (0.3 M phosphoric acid). Separation was conducted using a Synergi-C18 column (Phenomenex) and a mobile-phase gradient of buffer A (50 mM potassium phosphate in water, pH 6.2) and buffer B (100% methanol), starting a gradient to increase the mobile phase B from 10% to 30% over 5 min, reducing the mobile phase B from 30% to 10% over 30 s and holding the mobile phase B at 10% for 7.5 min at a flow rate of 1 mL/min. SAH peptide was monitored by UV absorbance at 260 nm and quantitated against a 4-point linear calibration curve produced with external SAH standards (Figure 1). Results were reported as pmol isoAsp/pmol protein. To enhance the detection of isoAsp in TLR9 adjuvanted vaccines, RFP-TLR-9 samples were treated with 0.2 M phosphate buffer prior to isoaspartic acid.

### 2.3. PcpA Protein Concentration Quantification by Reversed-Phase High-Performance Liquid Chromatography

To study the chemical degradation of PcpA under stressed conditions, the adjuvanted formulations were incubated at 45 °C for 5 weeks, and the concentration of intact protein was measured by RP-HPLC, as described elsewhere [16]. Briefly, samples were desorbed from the adjuvant and analyzed using an Agilent 1200 HPLC system equipped with a diode array UV detector and an ACE C4 column (Advanced Chromatography Technologies, Aberdeen, England). The separation was conducted using a mobile-phase gradient of buffer A (0.1% trifluoroacetic acid (TFA) in water) and buffer B (0.1% TFA in acetonitrile), 0.75% of buffer B per min over 30 min at a flow rate of 1 mL/min. Detection was performed by UV absorbance at 210 nm and quantitated against a 5-point linear calibration curve of PcpA standard.

### 2.4. Stability Studies

Formulations of recombinant proteins were prepared by mixing each individual antigen with aluminum salt adjuvant or TLR-9 agonist at room temperature under gentle mixing for 30 min. To assess the stability, formulations were incubated at 5 °C, 25 °C, 37 °C, 45 °C, and 55 °C. The stability of each antigen was evaluated as a function of time by isoaspartic acid concentration, as described above. Kinetic analysis was conducted using AKTS software version 5.42, as described elsewhere [17].

### 2.5. Statistical Analyses

Statistical analyses were performed using GraphPad Prism 10.2 software. Comparisons between two groups were performed using unpaired Student’s *t*-tests. The slopes were compared using analysis of covariance (ANCOVA). Comparisons of the nonlinear trends were performed using *F*-tests. 

## 3. Results and Discussion

### 3.1. IsoAsp Levels Are Increased in Thermally Stressed Vaccine Antigens

Deamidation is one of the most common chemical degradation pathways for proteins and peptides and has been shown to impact their chemical and biological properties [18]. Protein deamidation can affect protein folding, enzymatic activity, and degradation and has also been reported to impact the immunogenicity in protein-based vaccines against anthrax [9]. Our initial investigation focused on whether the quantification of isoAsp can be used to determine the degree of deamidation and/or isomerization in various vaccine candidates. Heat stress is one of the most common factors leading to protein deamidation. To investigate whether the levels of isoAsp increase with temperature stress, we analyzed two recombinant proteins and one viral-based vaccine under different temperature-stressed conditions and storage times. When compared to their respective controls, samples of RFP and PcpA protein antigens and of IPV incubated at 45 °C each displayed an increase in isoAsp (Table 1). Taken together, the results suggest that the determination of isoAsp can be utilized to identify deamidation in proteins and inactivated viruses. This method is easier and faster than traditional methods such as MS. MS is normally employed in the identification of deamidation in proteins with respect to deamidation species and identification of deamidation sites [11]. While MS is a powerful technique for characterizing protein deamidation, it lacks the throughput needed for handling the high number of samples generated during stability studies and formulation development. Moreover, excipients, detergents, and adjuvant systems commonly used in vaccines often interfere with MS, necessitating multi-step sample preparation before analysis, which adds complexity to the analytical development and testing process [6]. Another issue observed in the determination of post-translational modifications in vaccines using MS is the poor sequence coverage of proteins adsorbed to aluminum salt adjuvants. This is hypothesized to result from poor desorption of the protein from the adjuvant [19]. The quantification of isoAsp has the potential to enable a rapid and direct evaluation of deamidation levels in vaccine antigens. Nevertheless, a pivotal consideration in this endeavor pertains to the possibility of interference by vaccine adjuvants on the accuracy of the assay.

### 3.2. IsoAsp Can Be Accurately Detected in Adjuvanted Vaccines

**Figure 2:**
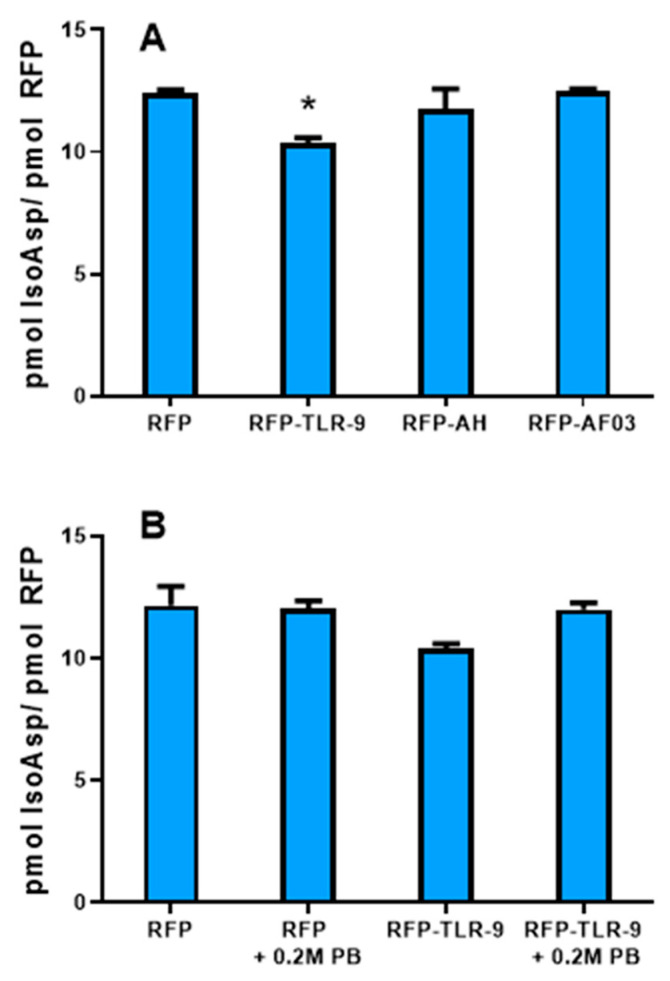
Quantification of isoaspartic acid (isoAsp) in adjuvanted vaccines. (**A**) IsoAsp quantification in a thermally stressed recombinant fusion protein (RFP) formulated either with toll-like receptor (TLR) 9 adjuvant, aluminum hydroxide (AH), or AF03 adjuvants. Lower recovery was observed in the recombinant fusion protein–toll-like receptor 9 adjuvant (RFP-TLR-9 adjuvant) formulation (* n = 3, *p* = 0.0002, unpaired *t*-test). (**B**) Increased recovery of isoAsp by treating RFP-TLR-9 adjuvanted with 0.2 M phosphate buffer (PB). No significant differences were observed in the isoAsp concentration when comparing RFP-TLR-9 adjuvant + 0.2 M PB versus RFP control (n = 3, *p* = 0.7403, unpaired *t*-test).

Adjuvants are typically added to vaccines to enhance immunogenicity and increase the durability of the immune responses [4]. Thus, we conducted an investigation to determine the reliability of isoAsp quantification in the presence of commonly used vaccine adjuvants. This investigation aims to ensure the robustness and applicability of the method in the context of vaccine development and analysis, where the presence of adjuvants is commonplace. In addition, the potential detection of isoAsp in adjuvanted vaccines without any major sample manipulations would improve and hasten the detection of deamidation during formulation development and stability evaluation of vaccines. To investigate whether isoAsp can be detected in the presence of adjuvants, the model protein RFP was first thermally stressed at 37 °C and then combined with a TLR9 agonist, aluminum oxyhydroxide, or a squalene-emulsion-based AF03 adjuvant. If the detection of isoAsp is not affected by the presence of adjuvants, similar levels of isoAsp should be observed in adjuvanted and non-adjuvanted samples. At the incubation temperature of 37 °C, the isoAsp levels in samples adjuvanted with aluminum hydroxide and AF03 were comparable to that of the unadjuvanted control sample (Figure 2A). These results indicate that the deamidation via isoAsp quantification can be directly monitored in samples adjuvanted with either AlOOH or AF03. However, in the RFP-TLR9 adjuvant formulation, a significant decrease (*p* = 0.0002) in the level of isoAsp was observed when compared to the unadjuvanted RFP control (Figure 2A). This result suggests that the TLR9 adjuvant may potentially interfere with the detection of deamidated Asp or Asn residues, possibly due to a strong electrostatic interaction with RFP. Introducing phosphate ions could reduce the electrostatic attraction between RFP and the TLR9 adjuvant, thereby facilitating the detection of isoAsp. To test this hypothesis, we explored whether the addition of phosphate ions could mitigate this interference. As shown in Figure 2B, the addition of phosphate did make a significant difference between the isoAsp levels of TLR9 adjuvanted and unadjuvanted samples. These results suggest a pivotal role for phosphate ions in enhancing the detection of isoAsp in TLR9 adjuvanted vaccines, potentially disrupting the electrostatic interactions between the antigen and the TLR9 adjuvant. This finding suggests that a simple sample treatment with phosphate ions could help mitigate interferences and improve the accuracy of isoAsp detection.

**Figure 3:**
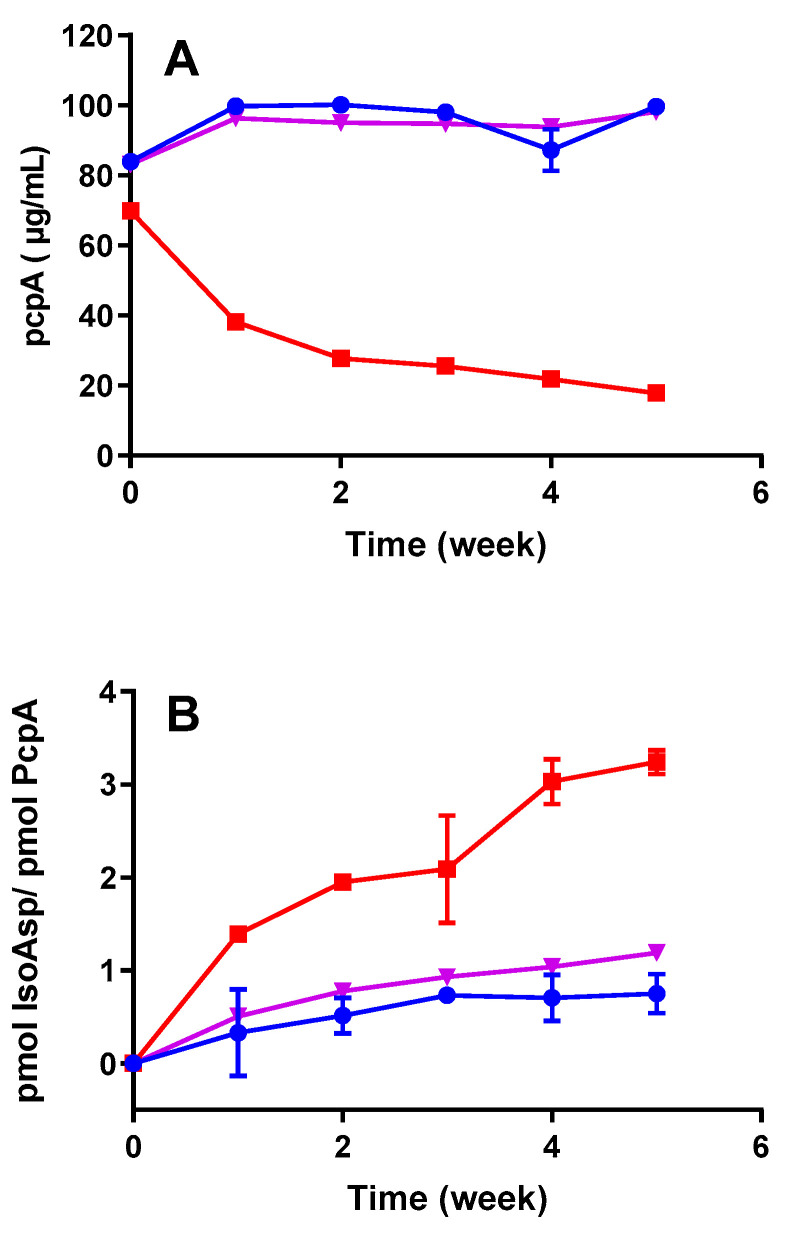
Effect of phosphate treatment of AlOOH on the stability of PcpA as evaluated by RP-HPLC (**A**) and isoAsp acid quantification (**B**). PcpA alone (blue circle), PcpA with AH (red square), and PcpA with phosphate-treated aluminum hydroxide (PTAH) 1.0 (purple triangle) were incubated for 5 weeks at 45 °C and tested weekly. Error bars represent the standard deviation from the mean (n = 3). The slopes obtained for PcpA with AH (red squares) were significantly higher than those obtained for PcpA in PTAH (purple triangles) or PcpA alone (blue circles) (ANCOVA, *p* < 0.0001).

When developing vaccines, determining the amount of degradation that occurs over time is critical in assessing the stability and setting shelf-life. Comparing the results of the isoAsp detection with known stability-indicating methods such as RP-HPLC could provide valuable insight into the application of isoAsp quantification. Previously, it has been shown that PcpA is sensitive to the high microenvironment pH of AlOOH, which causes protein instability, and thus, the stability of PcpA in AlOOH was improved with the addition of phosphate ions [16]. We investigated whether deamidation was involved in the decreased stability of PcpA exposed to high microenvironment pH by quantifying the amount of isoAsp in the formulation. Thus, we compared the stability profile of PcpA adjuvanted with AlOOH, phosphate-treated AlOOH (PT-AlOOH), or the unadjuvanted control. The RP-HPLC results were evaluated and compared to the isoAsp levels for PcpA antigen incubated at 45 °C. Compared to the unadjuvanted control, PcpA in AlOOH shows a higher degradation rate by HPLC when stored at 45 °C (Figure 3A), which correlated with an increase in isoAsp concentration (Figure 3B). PcpA adjuvanted with PT-AlOOH did not show a drastic change by RP-HPLC or an increase in isoAsp levels. These results indicate that treatment of AlOOH with phosphate led to a decrease in the microenvironment pH, which slowed down the rate of PcpA deamidation at high temperatures. Furthermore, the results suggest that isoAsp quantification can be used to assess deamidation, a possible mechanism of degradation linked to the stability of adjuvanted vaccine antigens and shelf-life determination. Consistent with our results, peptide mapping and mass spectrometry (MS) analyses have previously revealed that protein antigens, when bound to AlOOH, exhibited more susceptibility to deamidation reactions [6]. These findings emphasize the significance of understanding the impact of adjuvants on the stability and post-translational modifications of vaccine antigens, shedding light on potential challenges in vaccine development and formulation.

### 3.3. IsoAsp Levels in Vaccines Are Impacted by pH and Temperature 

**Figure 4:**
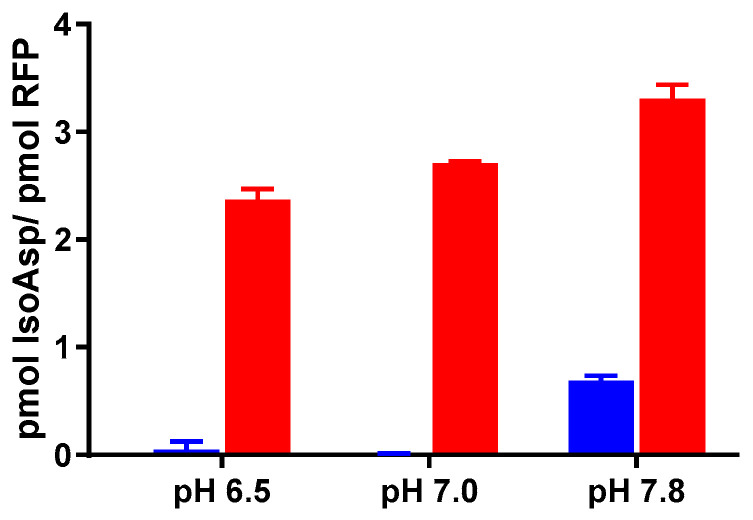
Effect of pH and stress temperature of 55 °C on deamidation rate of RFP-TLR-9 adjuvant. IsoAsp was measured at time zero (blue bars) and after 2 weeks of incubation at 55 °C (red bar) as a function of formulation pH. Error bars represent the SD from the mean (n = 3). A significant increase in the isoAsp concentration was observed at pH 7 and pH 7.8 when compared to pH 6.5 (unpaired *t*-test, *p* < 0.05).

During the vaccine development process, the optimization of the formulation pH is imperative to ensure the stability of the final product [20]. It is widely recognized that pH not only influences the physical stability and folding of antigens but also exerts a significant impact on protein deamidation [5,8]. Achieving the optimal pH conditions not only preserves the structural integrity of vaccine antigens but also plays a crucial role in minimizing undesirable post-translational modifications, such as deamidation, which can compromise vaccine efficacy and safety [21,22]. We investigated whether the quantification of isoAsp levels could serve as a screening tool for identifying optimal pH conditions that minimize deamidation in a vaccine formulation. For this purpose, we employed the RFP-TLR9 adjuvant as a model antigen–adjuvant formulation and incubated it at 55 °C for 2 weeks at three different pH levels (Figure 4). Interestingly, the non-stressed samples under slightly alkaline pH (7.8) conditions exhibited higher levels of isoAsp compared to those maintained at neutral or slightly acidic pH (6.5) levels. Furthermore, under stressed conditions, we observed a significant pH-dependent increase in isoAsp levels (Figure 4). These findings emphasize the practicality of determining isoAsp concentration as a valuable tool to assess formulation pH conditions that mitigate deamidation. They also underscore the critical importance of pH optimization in vaccine development to preserve the integrity of vaccine products.

**Figure 5:**
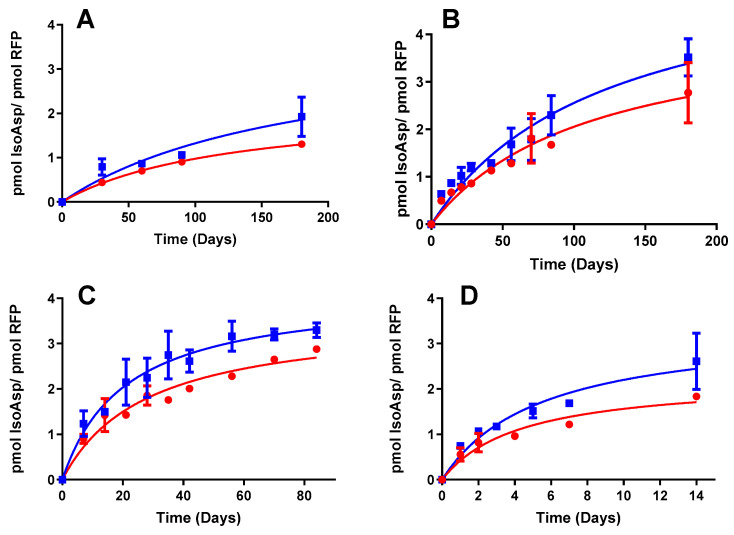
IsoAsp quantification ability to monitor long-term deamidation as a function of pH. IsoAsp was measured for RFP-TLR-9 adjuvant at pH 6.5 (red) and pH 7.8 (blue) as a function of time at 25 °C (**A**), 37 °C (**B**), 45 °C (**C**), and 55 °C (**D**). Error bars represent the standard deviation from the mean (n = 3). Comparisons of the nonlinear trends were performed using *F*-tests; statistically significant differences were observed among the trends obtained at pH 6.5 and 7.8 (*p* < 0.01).

To confirm whether the screening results align with long-term product stability, we subjected formulations at pH 6.5 and pH 7.8 to various temperatures and monitored isoAsp levels over time. Interestingly, we observed a significantly faster increase in isoAsp concentration over time in the pH 7.8 formulation compared to the pH 6.5 formulation across all temperature conditions (Figure 5A–D). These results are aligned with the screening experiments demonstrating a reduced deamidation rate of the RFP-TLR9 adjuvant at pH 6.5. At lower pH values, deamidation may be suppressed because the protonation of the nitrogen atom in the amide group can make it difficult for water to attack the carbonyl carbon and initiate the deamidation reaction [8]. While this paper does not specifically establish a direct correlation between deamidation and the activity or potency of the vaccine drug product, it is important to recognize that deamidation has the potential to significantly influence the antigenic properties of vaccine components [9]. As vaccines rely on the presence of specific epitopes to stimulate the immune system, any modifications induced by deamidation, whether in structure, conformation, or antibody binding properties, can lead to a diminished immune response or even a loss of efficacy [9]. The significance and impact of deamidation may vary depending on the specific antigen. Nevertheless, it should consistently be regarded as a potential critical quality attribute and assessed rigorously during the formulation development and stability testing phases. The determination of isoAsp could offer an economical and practical means to achieve this goal.

### 3.4. Thermal Kinetic Modeling Can Predict the Effects of pH and Temperature on IsoAsp Levels during Long-Term Storage

Accelerated stability programs, which expose products to temperatures greater than the recommended storage conditions, are typically carried out to estimate vaccine degradation rates [4]. Often, simple kinetic models are used to fit experimental data to enable prediction of a vaccine product’s shelf-life. However, vaccines, like other biological products, do not always follow simple kinetics models such as zero- or first-order kinetics. Using advanced thermokinetic data analysis with kinetic parameters derived from two-step models can help predict the complex decomposition dynamics of biological materials (19). AKTS modeling was performed to compare the shelf-life of two formulations prepared at pH 6.5 and 7.8 during a long-term stability study conducted under normal and various stressed conditions (5 °C, 25 °C, 37 °C, 45 °C, and 55 °C). 

**Figure 6:**
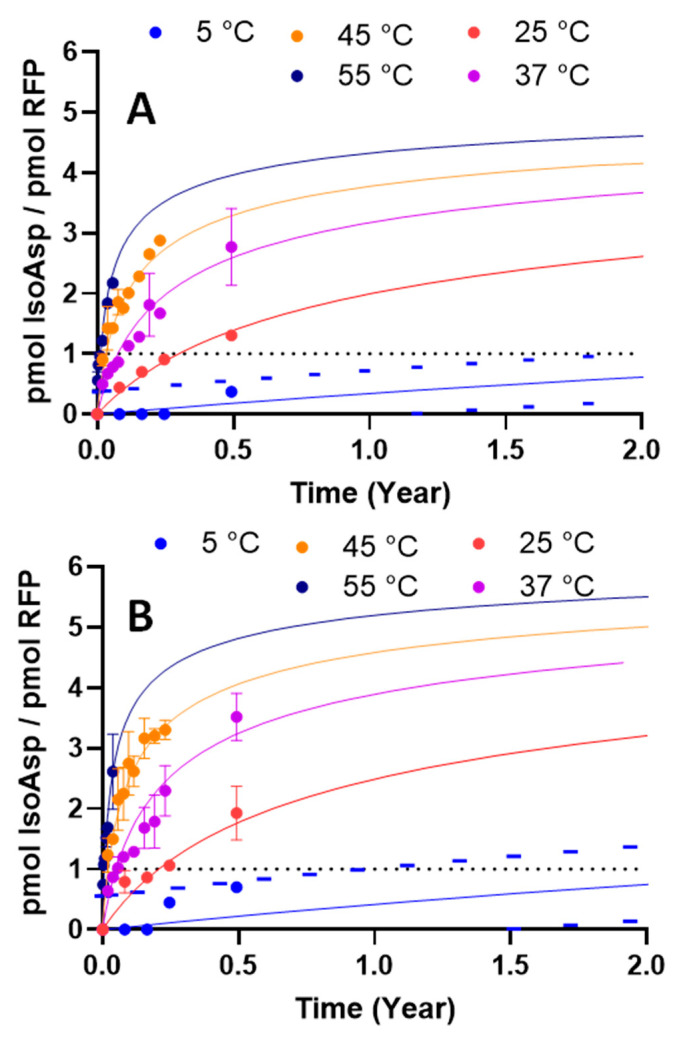
The long-term stability of RFP-TLR-9 adjuvant indicates that deamidation, monitored by isoAsp, is minimized at pH 6.5. Kinetic prediction modeling for long-term stability for pH 6.5 (**A**) and 7.8 (**B**) was performed by analyzing the data obtained at 5 °C, 25 °C, 37 °C, 45 °C, and 55 °C. Error bars represent the SD from the mean (n = 3). Prediction parameters are reported in Table 2.

The best kinetic model curves that fit the experimental data are presented in Figure 6. The prediction model demonstrated that the stability of the RFP-TLR9 adjuvant at pH 6.5 was greater than that at pH 7.8 (Figure 6A,B). To compare the stability of the two formulations, the shelf-life was calculated as the time at which the 95% confidence interval intercepts the arbitrarily pre-determined upper limit of 1 pmol of isoAsp/pmol of RFP (Table 2). Thus, the RFP-TLR9 adjuvant stored at a slightly acidic pH was predicted to take 1.9 years at 5 °C to meet the upper limit. In comparison, the formulation at pH 7.8 was predicted to reach the upper limit 9 months earlier in normal storage conditions (1.03 years at 5 °C). These results indicate that data generated from the quantification of isoAsp can be used to predict the stability of drug products. As deamidation is a type of degradation pathway known to impact the stability of vaccines, selecting a formulation that extends the vaccine’s shelf-life results in a potentially more robust and efficacious marketable vaccine.

## 4. Conclusions

Deamidation, a post-translational chemical modification occurring within protein-based vaccines, is a critical factor that can significantly impact vaccine quality and efficacy. This process is influenced by a variety of factors, including protein structure, temperature, and pH levels. The rate at which deamidation reactions occur can have far-reaching consequences, as they can lead to detrimental changes in the structure, function, stability, and immunogenicity of the vaccine. In vaccine development, the occurrence of deamidation is generally considered undesirable, making its detection and quantification essential. However, it is often challenging due to the presence of adjuvants and the lack of straightforward, quantitative techniques. Herein, we addressed this challenge by applying a simple yet effective method for quantifying isoAsp, which enables the easy detection and quantification of deamidation in vaccines. The results in this study highlight the significance of quantifying isoAsp as a screening tool to optimize vaccine formulations. High levels of isoAsp were detected in adjuvanted vaccines subjected to thermal and pH-induced stress, indicating significant deamidation. This finding emphasizes the potential of the assay to serve as a stability-indicating tool for vaccines. This study further identifies deamidation as a contributing factor to the degradation of proteins adsorbed to AlOOH, likely due to the high microenvironment pH associated with this adjuvant. 

Furthermore, the quantification of isoAsp in stability programs of a vaccine drug product allowed us to establish thermal kinetic modeling that could be used to predict deamidation over time with potential applications to product shelf-life determination. 

Altogether, this study presents a rapid and straightforward method for detecting and quantifying deamidation in adjuvanted vaccines, which can be applied to various aspects of vaccine development, including formulation screening, formulation optimization, stability assessment, and shelf-life determination. By offering a reliable tool for identifying deamidation early in the development process, this research contributes to ongoing efforts to optimize vaccine quality, stability, and safety.

## Figures and Tables

**Table 1:** IsoAsp levels measured in thermally stressed antigens.

Antigen and Stress Condition	Iso-Asparatate (Pmole/µg of Antigen) (Control Stored at −20 °C)	Iso-Asparatate (Pmole/µg of Antigen) (Thermally Stressed *)
RFP (37 °C)	2.71	12.31 *
PcpA antigen (45 °C)	Not detected	0.49
IPV antigen (45 °C)	Not detected	27.82

* Statistically significant, *p* = 0.0027, unpaired *t*-test (n = 3).

**Table 2:** Arrhenius equation summary for AKTS modeling.

Formulation pH	Kinetic Model	wAIC	wBIC	RSS	ln(A·s)	E (kJ/mol)	N (Reaction Order)	Time to Reach Upper Acceptance Criteria
pH 6.5	One step	71.46	71.46	2.48	18.03	87.84	4	1.90 years
pH 7.8	One step	61.26	61.26	5.55	19.66	91.51	4	1.03 years

Acronyms: wAIC = weighted Akaike information criterion; wBIC = weighted Bayesian information criterion; RSS = residual sum of squares; A = pre-exponential factor from Arrhenius equation.

## Data Availability

All the data that support this study are available from the corresponding author upon reasonable request.
